# Increased miltefosine tolerance in clinical isolates of *Leishmania donovani* is associated with reduced drug accumulation, increased infectivity and resistance to oxidative stress

**DOI:** 10.1371/journal.pntd.0005641

**Published:** 2017-06-02

**Authors:** Deepak Kumar Deep, Ruchi Singh, Vasundhra Bhandari, Aditya Verma, Vanila Sharma, Saima Wajid, Shyam Sundar, V. Ramesh, Jean Claude Dujardin, Poonam Salotra

**Affiliations:** 1 National Institute of Pathology (ICMR), Safdarjung Hospital Campus, New Delhi, India; 2 Department of Biotechnology, Faculty of Science, Jamia Hamdard, New Delhi, India; 3 Institute of Medical Sciences, Banaras Hindu University, Varanasi, India; 4 Dermatology Department, Safdarjung Hospital and Vardhman Mahavir Medical College (VMMC), New Delhi, India; 5 Unit of Molecular Parasitology, Department of Parasitology, Institute of Tropical Medicine, Antwerp, Belgium; Institute of Postgraduate Medical Education and Research, INDIA

## Abstract

**Background:**

Miltefosine (MIL) is an oral antileishmanial drug used for treatment of visceral leishmaniasis (VL) in the Indian subcontinent. Recent reports indicate a significant decline in its efficacy with a high rate of relapse in VL as well as post kala-azar dermal leishmaniasis (PKDL). We investigated the parasitic factors apparently involved in miltefosine unresponsiveness in clinical isolates of *Leishmania donovani*.

**Methodology:**

*L*. *donovani* isolated from patients of VL and PKDL at pretreatment stage (LdPreTx, n = 9), patients that relapsed after MIL treatment (LdRelapse, n = 7) and parasites made experimentally resistant to MIL (LdM30) were included in this study. MIL uptake was estimated using liquid chromatography coupled mass spectrometry. Reactive oxygen species and intracellular thiol content were measured fluorometrically. Q-PCR was used to assess the differential expression of genes associated with MIL resistance.

**Results:**

LdRelapse parasites exhibited higher IC_50_ both at promastigote level (7.92 ± 1.30 μM) and at intracellular amastigote level (11.35 ± 6.48 μM) when compared with LdPreTx parasites (3.27 ± 1.52 μM) and (3.85 ± 3.11 μM), respectively. The percent infectivity (72 hrs post infection) of LdRelapse parasites was significantly higher (80.71 ± 5.67%, P<0.001) in comparison to LdPreTx (60.44 ± 2.80%). MIL accumulation was significantly lower in LdRelapse parasites (1.7 fold, P<0.001) and in LdM30 parasites (2.4 fold, P<0.001) when compared with LdPreTx parasites. MIL induced ROS levels were significantly lower (p<0.05) in macrophages infected with LdRelapse while intracellular thiol content were significantly higher in LdRelapse compared to LdPreTx, indicating a better tolerance for oxidative stress in LdRelapse isolates. Genes associated with oxidative stress, metabolic processes and transporters showed modulated expression in LdRelapse and LdM30 parasites in comparison with LdPreTx parasites.

**Conclusion:**

The present study highlights the parasitic factors and pathways responsible for miltefosine unresponsiveness in VL and PKDL.

## Author summary

Increasing rate of relapse against miltefosine (MIL) and decline in its efficacy prompted us to study the parasitic factors associated with MIL unresponsiveness in clinical isolates of *Leishmania donovani*. Studies to explore the mechanism of MIL resistance in *L*. *donovani* are largely restricted to experimentally induced resistant parasites. In the present study, parasites isolated from MIL treated patients that relapsed (LdRelapse) were found to exhibit increased metacyclogenesis and infectivity to macrophages, decreased miltefosine accumulation and increased tolerance towards MIL induced oxidative stress in comparison to isolates from pretreatment cases (LdPreTx). Reduction in drug accumulation and increase in intracellular thiol content as well as tolerance to MIL induced oxidative stress were the highest in experimentally induced MIL resistant parasites (LdM30). Both LdRelapse and LdM30 parasites showed differential expression of genes associated with oxidative stress, metabolic processes and transport activity in comparison with LdPreTx parasites. The present study revealed that the parasites isolated from the cases that relapsed exhibited high infectivity, increased metacyclogenesis, reduced drug accumulation and reconfigured metabolism to overcome the oxidative stress induced during MIL exposure. These factors may be contributing to the high relapse rate observed in MIL treated VL and PKDL patients.

## Introduction

Visceral leishmaniasis (VL) is the most severe form of leishmaniasis, being fatal if left untreated. More than 90% of the global burden of VL occurs in just six countries: India, Bangladesh, Sudan, South Sudan, Brazil and Ethiopia [1]. India alone shares almost 50% of the world’s VL burden with Bihar, Uttar Pradesh, West Bengal and Jharkhand as the endemic states. Post kala-azar dermal leishmaniasis (PKDL), a dermal sequel reported in 5–15% of VL treated cases in the Indian subcontinent is reported to serve as a major reservoir of *Leishmania* [2,3].

Miltefosine (MIL) was introduced in the Indian subcontinent for VL treatment with cure rate of more than 94% [4]. However, the relapse rate has increased to 10% in India [5] and even more in Nepal where it ranged from 10% to 20% during 6 month and 12 month follow up, respectively [6]. The relapse rate in PKDL has also increased substantially (from 4% to 15%) [3]. The mean MIL IC_50_ value of the parasites obtained from pretreatment cases (often referred to as MIL sensitive cases) is lower in comparison to mean MIL IC_50_ value of the parasites obtained from the relapse cases in both Indian (VL and PKDL) and Nepalese (VL) patients [3, 5–8]. Increased infectivity and metacyclogenesis of parasites have been linked with high relapse rate in MIL treated Nepalese patients [6]. Emergence of MIL tolerant parasites due to prolonged exposure to MIL may be associated with decline in drug efficacy, as observed in case of antimony resistance [3, 9].

Experimental resistance to MIL was shown to be readily induced *in vitro* [10–12]. Impairment in drug uptake machinery involving amino-phospholipid translocase miltefosine transporter (LdMT) and an accessory protein (LdRos3) was proposed to be the most likely mechanism of resistance [10–13]. Recent study from our group on comparative transcriptome profiling of MIL sensitive and laboratory generated resistant parasites revealed altered expression of genes involved in thiol metabolism, drug transport, protein translation and folding, DNA repair and replication machinery [12]. Miltefosine tolerant parasites of *L*. *donovani* exhibited greater ability to resist reactive oxygen species than the sensitive parasites [14]. Suppression of oxidative stress induced apoptotic cell death has been reported in MIL resistant *L*. *donovani* parasites [15].

Majority of evidence regarding parasitic factors associated with the development of miltefosine resistance is restricted to laboratory adapted resistant parasites and limited information is available with respect to clinical isolates of *L*. *donovani* [10–12]. Understanding the parasitic factors and molecular mechanisms involved in miltefosine unresponsiveness in clinical isolates of *L*. *donovani* is crucial to monitor drug efficacy and its longevity. In the present study, we have investigated several parasitic factors including *in vitro* MIL susceptibility, infectivity to macrophages, metacyclogenesis, drug accumulation, intracellular thiol content, reactive oxygen species (ROS) and targeted transcript profile of genes implicated in MIL resistance, in parasites obtained from the cases of VL and PKDL that relapsed (LdRelapse) in comparison to the parasites from pretreatment cases (LdPreTx), and laboratory adapted MIL resistant parasites (LdM30).

## Materials and methods

### *L*. *donovani* isolates

Clinical isolates of *L*. *donovani* used here were reported in our previous studies [3, 7, 12]. Parasite isolates were prepared from splenic aspirates of VL patients or from dermal lesions of PKDL patients reporting to KAMRC, Muzaffarpur, Bihar or Safdarjung Hospital (SJH), New Delhi, under the guidelines of the Ethics Committee of the respective Institutes. Parasites were isolated from each of the relapse cases at the time of reported relapse (after completion of MIL treatment at four, six and seven months VL and at 12 and 18 months in PKDL) and were designated as MHOM/IN/year/code/month (at which relapse occurred). Experimental MIL resistant parasites were prepared as described earlier [12]. Briefly, wild type *L*. *donovani* promastigotes were adapted to grow under high MIL pressure by *in vitro* passage with stepwise increase in the MIL concentration (2.5, 5, 7.5,10, 20 and 30 μg/ml) in medium M199 to generate MIL resistant parasite (LdM30). At each step, parasites were cultured for at least 4–6 passages to attain steady growth comparable to the wild type counterpart. The susceptibility towards MIL of adapting parasites was assessed at each step during adaptation. Adapted parasites were maintained under miltefosine pressure (30 μg/ml) in M199 medium. LdAG83 parasite was used as standard reference strain. Parasites were routinely grown in Medium 199 (Sigma, St. Louis, MO USA) supplemented with 10% heat-inactivated foetal bovine serum (HI FBS, Gibco, USA), 100 IU/ml penicillin G, and 100 μg/ml streptomycin at 25°C. Mouse derived peritoneal macrophages were cultured in RPMI1640 medium supplemented with 10% FCS, 100 IU/ml penicillin G, and 100 μg/ml streptomycin at 37°C in 5% CO_2_.

### *In vitro* miltefosine susceptibility assay

Drug susceptibility of the parasites towards MIL was estimated at the promastigote stage as described previously [16]. Briefly, late log phase *L*. *donovani* promastigotes (10^5^ promastigotes/well) were seeded into 96 well culture plate containing MIL concentration ranging from 0.4 μM to 390 μM. After 72 h incubation at 25°C, 50 μl resazurin [0.0125% (w/v in PBS)] was added, and plates were incubated for a further 24 h. Cell viability was measured fluorometrically (λex 550 nm; λem 590 nm) on Infinite M200 multimode reader (Tecan). The results were expressed as percentage reduction in the parasite viability compared to untreated control wells. 50% inhibitory concentration (IC_50_) was calculated by sigmoidal regression analysis. All experiments were performed at least thrice in quadruplicate.

Similarly, *in vitro* MIL susceptibility was assessed at intracellular amastigote level by following macrophage-amastigote model described elsewhere [17] with modifications. Briefly, the mice peritoneal exudates derived macrophages were infected with late log phase parasites at a ratio of 10 parasites per macrophage, in a volume of 200 μl complete RPMI 1640 medium into 8-well chamber slides and incubated for 16 h at 37°C in 5% CO_2_. Excess, non-adhered promastigotes were removed by washing and infected cells were re-incubated for 48 h, with MIL (1, 5, 10, 20 and 40 μM). Macrophages were then examined for intracellular amastigotes after staining with Diff-Quik solutions. The number of *L*. *donovani* amastigotes was counted in 100 macrophages, at 1000x magnification. The IC_50_ was calculated using sigmoidal regression analysis. All experiments were performed at least thrice in duplicate.

### Infectivity to macrophages

The percentage of *L*. *donovani* infected macrophages was assessed following macrophage-amastigote model as described elsewhere with modifications [17–19]. Primary peritoneal macrophages were extracted from female BALB/c mice, plated at 2x10^5^ cells per well in RPMI 1640 medium supplemented with 10% FBS in 8-well chamber slides, and incubated at 37°C in 5% CO_2_. Twenty-four hours later, the medium was gently removed and the macrophages were infected with late log phase promastigotes at a ratio of 10 parasites per macrophage, in a volume of 200 μl complete RPMI 1640 medium without MIL. After 6 h of infection, non-internalized parasites were washed off, and after addition of fresh complete RPMI medium the slide was further incubated for 18 h (group 1), 42 h (group 2) and 66 h (group 3). Slides were fixed with methanol and stained with Diff-Quik solutions. A total of 500 macrophages were counted in randomly selected fields for each group at 1000x magnification. The percentage of infected macrophages was calculated by counting the number of infected cells out of 100 macrophages. The experiment was repeated thrice.

### Metacyclogenesis in *L*. *donovani*

Percent metacyclogenesis was assessed following the methods described elsewhere [20, 21]. Briefly, stationary phase promastigotes from LdRelapse (n = 7) and LdPreTx (n = 9) were harvested at 2000g X 10 min and resuspended at a cell density of 2X10^8^ cells/ml in 10 ml of complete M199 medium containing 50 μg/ml peanut agglutinin (PNA) (Sigma, St. Louis, MO USA). Promastigotes were allowed to agglutinate at room temperature for 30 min and the sediment and the supernatant were recovered. The sediment was diluted to the initial volume in fresh complete medium containing 50 μg/ml PNA. Both fractions were centrifuged at 200g for 10 min and the supernatant obtained were centrifuged at 2000g to obtain PNA^**-**^ (metacyclic) promastigotes. All steps were checked under a light microscope (Nikon ECLIPSE TS100). Percent metacyclic population was calculated by counting PNA^**-**^ population out of the total promastigote cell density.

### Miltefosine accumulation assay

MIL accumulation was studied according to the protocol described earlier, with slight modifications [22, 23]. Briefly, log phase promastigotes (10^8^ cells/ml) were incubated with 100 μM MIL for 90 min, washed twice with PBS and digested by overnight incubation in 200 μl of 2 N HNO_3_. The lysate was made up to 1 ml with PBS and analyzed for MIL content using liquid chromatography-mass spectrometry (LC-MS). Chromatographic separation of MIL was carried out using an HP1290 liquid chromatograph system (Agilent Technologies; Palo Alto, CA, USA) consisting of a binary pump, degasser and auto-sampler. Chromatography was performed on a ZORBAX Eclipse XDB C8 column, maintained at 45°C. A mobile phase of 0.1% formic acid in methanol and water (95:5; v/v) at a flow rate 0.45 ml/min was employed. The mass spectrometer detection system consisted of an AB sciex API 3000 LCMS with electron spray ionization (ESI) positive mode. The mass spectrometer was operated in MRM mode. The setting of the mass spectrometer was as follows: the spray voltage 5000 V, nebulizer gas flow 12.0 L/min, curtain gas flow 8.0 L/min, collision gas (Nitrogen) flow 6.0 L/min. The source temperature was 450°C. Declustering potential, focussing potential, entrance potential were 40, 150 and 10 V, respectively. The transition of 408.5 to 125.0 was optimised for MIL with 40 V collision energy. Data were processed using Analyst TM software (version 1.6.2; Sciex). The final concentration was calculated after multiplying the value obtained with dilution factor and presented in ng/ml.

### Preparation of standard curve

All stock and working solutions were prepared in methanol–water (1:1, v/v). MIL stock solution was prepared at a concentration of 0.1 mg/ml for the preparation of calibration standards. This solution was further diluted to obtain working solutions with concentrations of 0.5, 1.0, 2.5, 5.0, 10.0, 25.0, and 50 ng/ml. Calibration standards were prepared freshly before each analytical run.

### Measurement of miltefosine induced oxidative stress response

MIL induced oxidative stress response was studied both at the promastigote and the intracellular amastigote stage following the macrophage-amastigote model in terms of accumulation of reactive oxygen species (ROS) described elsewhere with modifications [24]. All the experiments were carried out in triplicate.

Promastigotes were exposed to MIL (5, 10, 15 and 20 μM) for 48 h and centrifuged at 3000 rpm for 10 min. Cells (2x10^7^ parasites/ml) were resuspended in Hepes–NaCl (21 mM Hepes, 137 mM NaCl, 5 mM KCl, 0.7 mM Na_2_HPO_4_.7H_2_O, 6 mM glucose, pH 7.4) and incubated with 40 nM of cell permeable fluorescent probe 2’ 7’-Dichlorodihydrofluorescin Diacetate (H_2_DCF-DA) (molecular probe) for 45 minutes in dark at 26°C. Although the H_2_DCF-DA has limitation as a probe for direct measurement of ROS [25], it has been widely used for assessment of ROS levels in *Leishmania* [24, 26]. Fluorescence was measured at 495 nm excitation and 535 nm emission wavelength using cytofluorimeter (Infinite M200, Tecan, Switzerland).

The mice peritoneal exudates derived macrophages were infected with LdPreTx, LdRelapse or LdM30 parasites in a ratio of 1:10 in 96 well tissue culture plates and incubated with 5% CO2−95% air mixture at 37°C in a CO_2_ incubator. After overnight infection, cells were washed with incomplete RPMI (without FBS) medium to get rid of the uninternalized promastigotes followed by addition of MIL at 20 μM. After 48 h of drug exposure, the cells were washed with incomplete RPMI, 30 μM of H_2_DCF-DA was added in 200 μl volume of incomplete RPMI and the plate was incubated further for 30 min. Uninfected macrophages with MIL exposure were taken as control to measure background fluorescence that was subtracted from the fluorescence measured at 495nm excitation and 535 nm emission wavelength (Infinite M200, Tecan, Switzerland). The fluorimetric measurements were expressed as mean fluorescent intensity units (MFI) that represented level of ROS.

### Intracellular thiol levels in *L*. *donovani* promastigotes

The intracellular thiol concentration was measured using thiol detection assay kit (Cayman Chemical Company, MI, USA) following manufacturer’s instructions. Briefly, 1x10^7^ late log phase promastigotes were harvested by centrifugation at 2000 g at 4°C for 10 min. After washing with 1x PBS (pH 7.4) cell pellet was homogenised in 0.5 ml cold buffer (100 mM Tris-HCI, pH 7.5, containing 1mM EDTA) and centrifuged at 10,000g for 15 min at 4°C. Supernatant was collected for the fluorometric estimation of thiol at 390 nm excitation and 520 nm emission wavelengths. Thiol concentration was estimated in 100 μl of supernatant distributed into 96 well plate from three independent preparations and calculated by using the equation obtained from the linear regression of the standard curve, substituting adjusted fluorescence value for each sample as follows

$$\text{Thiol concentration }\left({\text{nmol/10}}^{\text{7}}\text{promastigotes}\right)=\frac{\text{Adjusted sample florescence-}\left(\text{y intercept}\right)\;\times \text{ sample dilution}}{\text{slope}}$$

### RNA extraction and real-time PCR analysis

Total RNA was extracted from 10^8^ late log phase promastigotes using Trizol reagent according to manufacturer’s instructions. RNA clean up was performed using RNeasy Plus mini kit (Qiagen, Gaithersburg, MD, USA) as described by the manufacturer. The purified RNA was quantified using Nanodrop by estimating the absorbance at 260 and 280 nm. Briefly, first strand cDNA was synthesized from 5 μg of total RNA of *L*. *donovani* parasites using the Superscript II RNAse H reverse transcriptase enzyme (Invitrogen, Carlsbad, CA, USA) and Oligo dT primers (Fermentas, USA) according to the manufacturer’s protocol. Three independent RNA preparations were used for each Q-PCR experiment. Equal amounts of cDNA were run in triplicate and amplified in 25 μl reactions containing 1x Fast SYBR Green Mastermix (Applied Biosystems, USA), 100 ng/ml forward and reverse primers. The sequences of the primers for all the genes (n = 11; including 2 genes as internal control) amplified in the study are given in Table 1. Reactions were performed in triplicate in an ABI Prism 7500 (Applied Biosystems, CA, USA). The analysis of gene expression was performed using the 2^-ΔΔCT^ method. The data was presented as the fold change in the target gene expression in *L*. *donovani* parasites normalized to the internal control genes [27, 28] (GAPDH and α-Tubulin) and relative to the LdAG83 reference strain of *L*. *donovani*.

**Table 1:** List of target genes, their putative function and primer sequence used for amplification.

S.No	Gene name	Protein/gene ID (*L*. *infantum* data base)	Function	Primer sequence (5’-3’)	Product size (bp)
1	TRYP	Tryparedoxin peroxidase/LinJ.15.1120	Oxidoreductase activity	Fp-GCTTCAACGAGCTCAACT GC Rp-CCCTGTTTCTCCTCCAGCAC	178
2	Cytb5Red	Cytochrome b5 reductase/LinJ.15.0050	Oxidoreductase activity	Fp-ACGCCGTTCTTTGGGTACG Rp-TGCCTTCTGAGTCTCCCACC	100
3	PGMPUT	Phosphoglucomutase putative/Linj.21.0700	Carbohydrate metabolic process	Fp-CGGCGCTTTTATCTTGACGG Rp-TAGGTGCCGAGGGTGTGGAT	195
4	LPP	Lipase precursor like protein/LinJ.31.0870	Lipid metabolism	Fp-TTGGACTTCTGGCTCACGC Rp-AAGGCTGCTGTAAGCGCTG	179
5	TSH	Trypanothione synthetase putative/LinJ.23.0500	Thiol metabolism	Fp-CGAACACATGGACAAGCACG Rp-TGAGTCGGTATGCGTACGTG	168
6	MDRP	Multidrug resistance like protein/LinJ.24.1510	Transport activity	FP-CTGTACGACCCCAACGGCTATCAGACT Rp-GTGCTCCAGGAAGAGGTAGTACAGGCTCAC	195
7	ABCF2	ATP binding cassette subfamily F/LinJ.33.0340	Transport activity	Fp-CTCTGCACAGCCATTCGTAA Rp-CTCTTTTTCGAACTCCGCAC	174
8	TCP20	Chaperonin TCP20, Putative/LinJ.13.1400	Protein folding	Fp-AAACTAACCTTGGGCCTCGT Rp-GACCACACTCGTACTCCCGT	185
9	AAT19	Amino acid transporter 19, putative/LinJ.07.1340	Transport activity	Fp-CACCATGGTCGTGTTCTTTG Rp-CGGATTCGCCATCTCATAGT	178
10	GAPDH	Glyceraldehydes-3 phosphatedehydrogenase/LinJ.30.2990	Internal control	Fp-GAAGTACACGGTGGAGGC TG Rp-CGCTGATCACGACCTTCT TC	206
11	α-TUBULIN	Alpha tubulin/LinJ.13.1450	Internal control	Fp-CTACGGCAAGAAGTCCAAGC Rp-CAATGTCGAGAGAACGACGA	179

Abbreviations: FP, Forward primer; Rp, Reverse primer

### Statistical analysis

Statistical analysis of data was performed using Graph Pad Prism 5 software (GraphPad Software Inc., San Diego, CA, USA). Statistical significance was determined by Student’s t-test. Correlation was determined by Spearman’s rank test. P values <0.05 were considered significant.

### Ethics statement

The study was approved by the Ethical Committee of the Institute of Medical Sciences, Banaras Hindu University (Dean 2007-08/42, dated 15-05-2008), Varanasi and Institutional Ethics committee of Safdarjung Hospital & VMMC (VMMC/SJH/PROJECT/22-10-2012/7), New Delhi, India. Written informed consent was obtained from patients. Clinical isolates of *L*. *donovani* were analyzed anonymously. *In vitro* susceptibility experiments and ROS tolerance at amastigote level were carried out using mice peritoneal derived macrophages after approval from Institutional Animal Ethics Committee (IAEC-3/2010) of National Institute of Pathology, New Delhi following guidelines for animal care and handling protocols recommended by committee for the purpose of control and supervision of experiments on animals (CPCSEA).

## Results

### *In vitro* drug susceptibility of *L*. *donovani* clinical isolates

**Fig 1:**
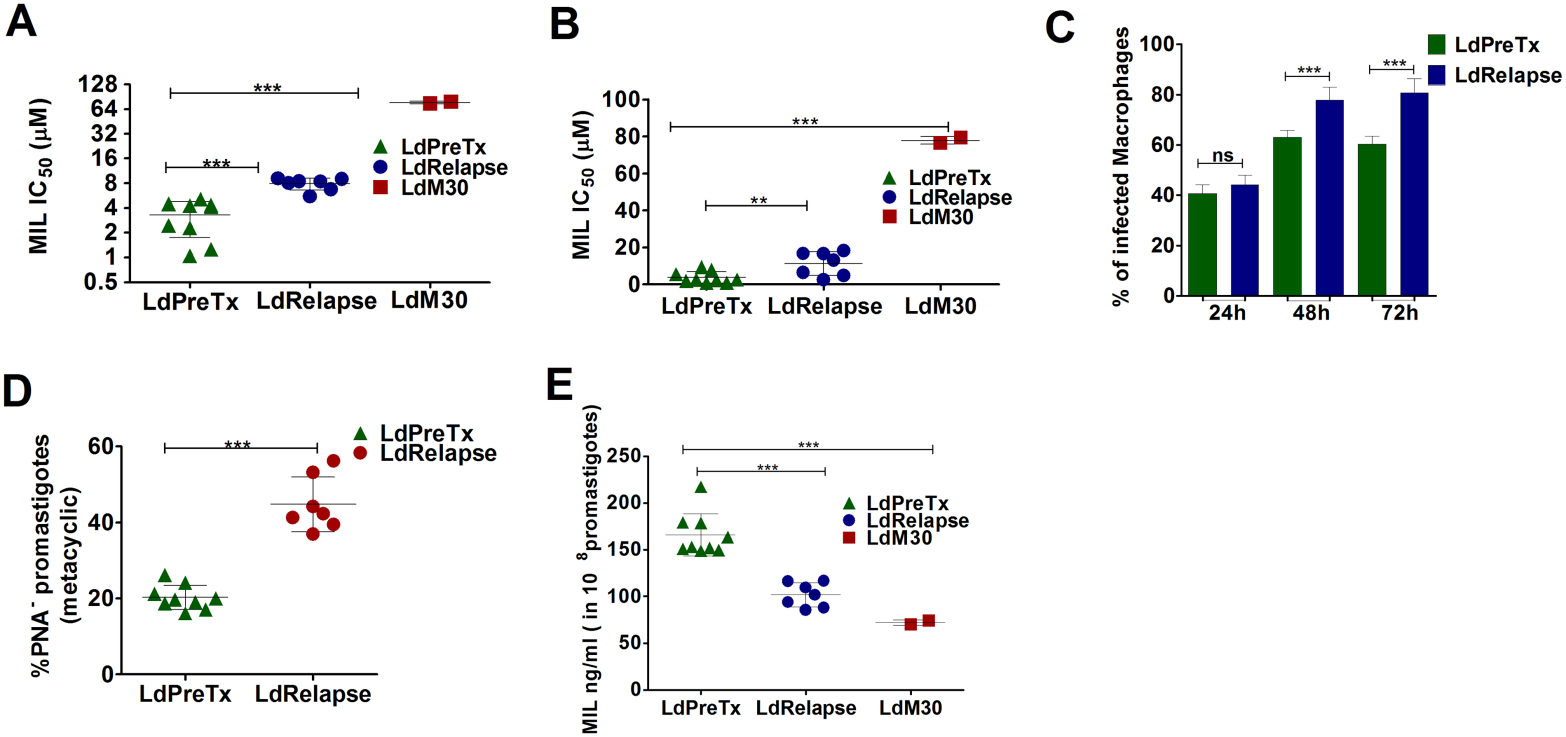
*In vitro* MIL susceptibility, percent infectivity and MIL uptake in *L*. *donovani* parasites. (A) MIL susceptibility at promastigotes stage of LdPreTx, LdRelapse and LdM30 with each individual value represented as mean IC_50_±SD from three separate assays. (B) MIL susceptibility at intracellular amastigote stage LdPreTx, LdRelapse and LdM30 with each individual value represented as mean IC_50_ ± SD from three separate assays (C) Mice peritoneal derived macrophages infected with LdPreTx or LdRelapse parasites at a 1:10 (cell/parasite) ratio. The percent infectivity was determined at 24h, 48h, and 72h post infection by counting number of infected cells out of 100 macrophages at 1000X magnification after staining with Diff-Quik. Data represents mean ± SD of three independent experiments each in duplicate. (D) Percent metacyclogenesis of promastigote population estimated based on negative selection of peanut agglutinin (%PNA^**-**^ promastigote). Values represent mean ± SD of two independent experiments (E) MIL uptake, estimated using LC-MS in 1x10^8^ promastigotes of LdPreTx, LdRelpase and LdM30. Data represents mean ± SD of two independent experiments, each in duplicate. Asterisks indicate significance (**P<0.01; and ***P<0.001).

MIL susceptibility profile of parasites isolated from pretreatment group LdPreTx (n = 9), from cases that relapsed after MIL treatment [VL (n = 3) and PKDL (n = 4)] along with experimental MIL resistant isolates LdM30 (n = 2) is given in Table 2. The mean IC_50_ at promastigote stage of LdRelapse group parasites (7.92 ± 1.30 μM) was significantly higher (P<0.001) than LdPreTx (3.27 ± 1.52 μM) while that of LdM30 parasites was the highest (76.50 ± 2.89μM) (Fig 1A). Likewise, the mean IC_50_ at intracellular amastigote stage of LdRelapse group parasites (11.35 ± 6.48 μM) was significantly higher (P<0.01) than LdPreTx (3.85 ± 3.11 μM) while that of LdM30 parasites was the highest (77.98 ± 2.00 μM) (Fig 1B).

**Table 2:** *In vitro* miltefosine susceptibility (IC_50_), MIL accumulation and thiol levels of *L*. *donovani* isolates from pretreatment group (LdPreTx), isolates obtained from visceral leishmaniasis (VL) and post kala-azar dermal leishmaniasis (PKDL) patients that relapsed after MIL treatment (LdRelapse) and experimental MIL resistant parasites (LdM30).

S.No.	Parasite	MILIC_50_±SD(μM) Promastigote	MIL IC_50_±SD(μM) Amastigote	Mean MILaccumulationng/ml (in 10^8^promastigotes)	Mean thiol in(nmol/10^7^promastigotes)
**LdPreTx VL**					
1	MHOM/IN/1983/AG83	**1.05±0.07**	**0.85±0.30**	**154.0±0.99**	**87.5 ± 13.4**
2	MHOM/IN/2010/BHU869/0	**4.35±0.21**	**1.05±0.08**	**221.0±1.06**	**102.0 ± 5.7**
3	MHOM/IN/2010/BHU902/0	**4.14±0.62**	**2.30±0.36**	**220.0±0.85**	**83.5 ± 3.5**
4	MHOM/IN/2010/BHU994/0	**4.48±1.10**	**1.96±0.50**	**165.4±0.28**	**77.4 ± 5.5**
5	MHOM/IN/2000/K133/0	**1.26±0.4**	**2.32±0.78**	**181.23±0.78**	**79.5 ± 0.7**
6	MHOM/IN/2009/BHU573/0	**2.45±0.7**	**2.45±0.30**	**180.56±0.07**	**74.5 ± 13.4**
**LdPreTx PKDL**					
7	MHOM/IN/2011/NIPP232/0	**4.28±0.38**	**7.99 ± 0.2**	**149.51±0.64**	**76.30 ± 0.65**
8	MHOM/IN/2012/NIPP251/0	**5.14±0.36**	**9.5 ± 1.2**	**148.84±0.35**	**89.23 ± 0.78**
9	MHOM/IN/2013/NIPP260/0	**2.27±0.01**	**5.65 ± 0.7**	**150.86±0.35**	**71.40 ± 0.45**
**LdRelapse VL**					
10	MHOM/IN/2010/BHU872/6	**9.14±0.69**	**4.84±0.39**	**094.80± 0.21**	**99.0 ± 2.2**
11	MHOM/IN/2009/BHU1062/4	**8.04±3.17**	**6.67±0.62**	**117.78 ± 0.07**	**130.6±10.4**
12	MHOM/IN/2010/BHU1113/7	**5.56±0.19**	**2.67±0.50**	**118.13 ± 0.42**	**129.0 ± 0.2**
**LdRelapse PKDL**					
13	MHOM/IN/2010/NIPP195/12	**8.50 ± 2.07**	**18.26±1.50**	**111.0 ± 0.07**	**126.0 ± 7.6**
14	MHOM/IN/2010/NIPP214/18	**6.76 ± 1.77**	**16.70±0.65**	**103.0 ± 0.07**	**114.0 ± 3.2**
15	MHOM/IN/2012/NIPP228/12	**9.00±1.40**	**16.80± 0.76**	**85.73±0.14**	**123.0 ± 0.2**
16	MHOM/IN/2012/NIPP229/18	**8.42±0.60**	**18.50±1.10**	**88.09±0.64**	**119.0 ± 0.4**
**LdM30**					
17	MHOM/IN/2000/K133M30	**78.60±3.12**	**79.40±2.35**	**74.93 ± 0.0**	**143.0 ± 8.1**
18	MHOM/IN/2009/BHU573M30	**74.50±2.38**	**76.56±1.92**	**70.88 ± 0.0**	**135.0 ± 9.9**

### Infectivity of *L*. *donovani* clinical isolates

The percentage of infected macrophages with parasites from LdRelapse group was significantly higher in comparison to the LdPreTx group at both 48 h (LdRelapse = 77.85 ± 5.17, LdPreTx = 60.11 ± 5.68, P<0.001) and 72 h post infection (LdRelapse = 80.71 ± 5.67, LdPreTx = 60.44 ± 2.80, P<0.001), although infectivity was comparable at 24h post infection (Fig 1C). LdM30 parasites exhibited similar infectivity as the wild type. This is as expected since these parasites were subjected to numerous *in vitro* passages during adaptation and continuous axenic cultivation of *Leishmania* promastigotes is known to lead to decreased infectivity [29].

### Increased metacyclogenesis in LdRelapse parasites

The percent meatcyclogenesis was evaluated based on negative selection with PNA in culture. LdRelapse parasites exhibited significant increase (P <0.001) in metacyclic promastigotes (PNA^**-**^) (44.81 ± 7.20%) when compared with LdPreTx (20.27 ± 3.19%) (Fig 1D). The increase in metacyclic population in LdRelapse correlated strongly (r = 0.92) with their infectivity to macrophages.

### Miltefosine accumulation in *L*. *donovani* clinical isolates

The mean MIL accumulation (ng/10^8^ promastigotes) in LdRelapse group parasites (102.6 ± 13.5) was significantly lower (1.7 fold, P<0.001) than that in LdPreTx (174.6 ± 28.9), while it was the lowest (2.4 fold) in LdM30 parasites (72.8 ± 2.8). We found a decline in MIL accumulation with increasing IC_50_ value (r = -0.78) (Table 2 and Fig 1E).

### ROS tolerance and intracellular thiol content in *L*. *donovani*

**Fig 2:**
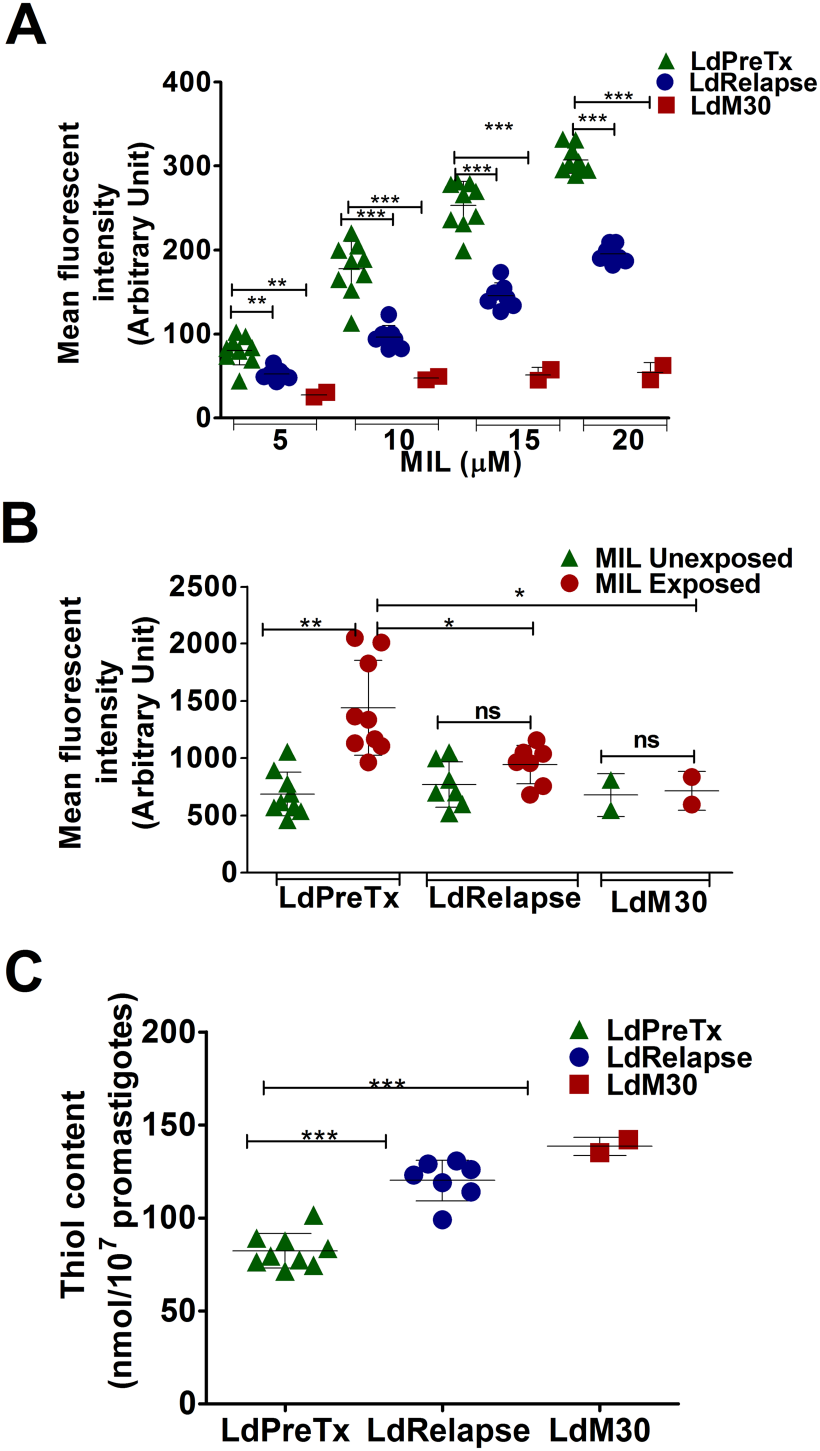
MIL induced oxidative stress (ROS level) and intracellular thiol content in *L*. *donovani*. (A) Dose dependent accumulation of ROS in LdPreTx, LdRelapse and LdM30 at promastigote stage was assayed fluorometrically at 495 nm excitation and 535 nm emission wavelength using cell permeable probe H_2_DCF-DA (40nM). Data represents mean ± SD of three independent experiments, each performed in triplicate. Asterisks indicate significance (**P<0.01; and ***P<0.001). (B) Accumulation of ROS in macrophages infected with LdPreTx, LdRelapse, LdM30 parasites before and after MIL exposure (20μM), assayed fluorometrically at 495 nm excitation and 535 nm emission wavelength using cell permeable probe H_2_DCF-DA (30μM). Data represents mean ± SD of three independent experiments, each in triplicate. Asterisks indicate significance (*P<0.05; **P<0.01; and ***P<0.001). (C) Intracellular thiol content in LdPreTx, LdRelapse and LdM30 promastigotes, measured fluorometrically at 390 nm excitation and 520 nm emission wavelength. Data represents mean ± SD of two independent experiments each performed in triplicate. Asterisks indicate significance (**P<0.01; and ***P<0.001).

At promastigote stage both LdPreTx and LdRelapse showed dose dependent increase in ROS level which LdM30 parasites did not. At 20 μM of MIL, the ROS level was significantly lower (P<0.001) in LdRelapse (MFI 195.28) compared to LdPreTx group (MFI 307.16). ROS level in LdM30 parasites (MFI 54.00) was significantly lower than both LdPreTx (P<0.001) and LdRelapse, (P<0.001) (Fig 2A). Subsequent to MIL exposure, macrophages infected with LdRelapse parasites or LdM30 showed no increase in ROS accumulation, while macrophages infected with LdPreTx group showed significant increase (≥2 fold, P<0.01) in ROS levels (Fig 2B). The tolerance to ROS was significantly (P<0.05) higher in MIL exposed macrophages infected with LdRelapse and LdM30 compared to LdPreTx.

The mean thiol content (nmol/10^7^ promastigotes) of LdRelapse (120.0 ± 1.1 nmol) was significantly (P<0.001) higher than that of LdPreTx (82.3 ± 9.3). The mean thiol content was the highest in LdM30 isolates (140.0 ± 5.5) (Fig 2C). Thiol content of *L*. *donovani* isolates strongly correlated (r = 0.78) with the IC_50_ value of the respective isolate.

### Targeted gene expression analysis in LdRelapse, LdPreTx and LdM30

**Fig 3:**
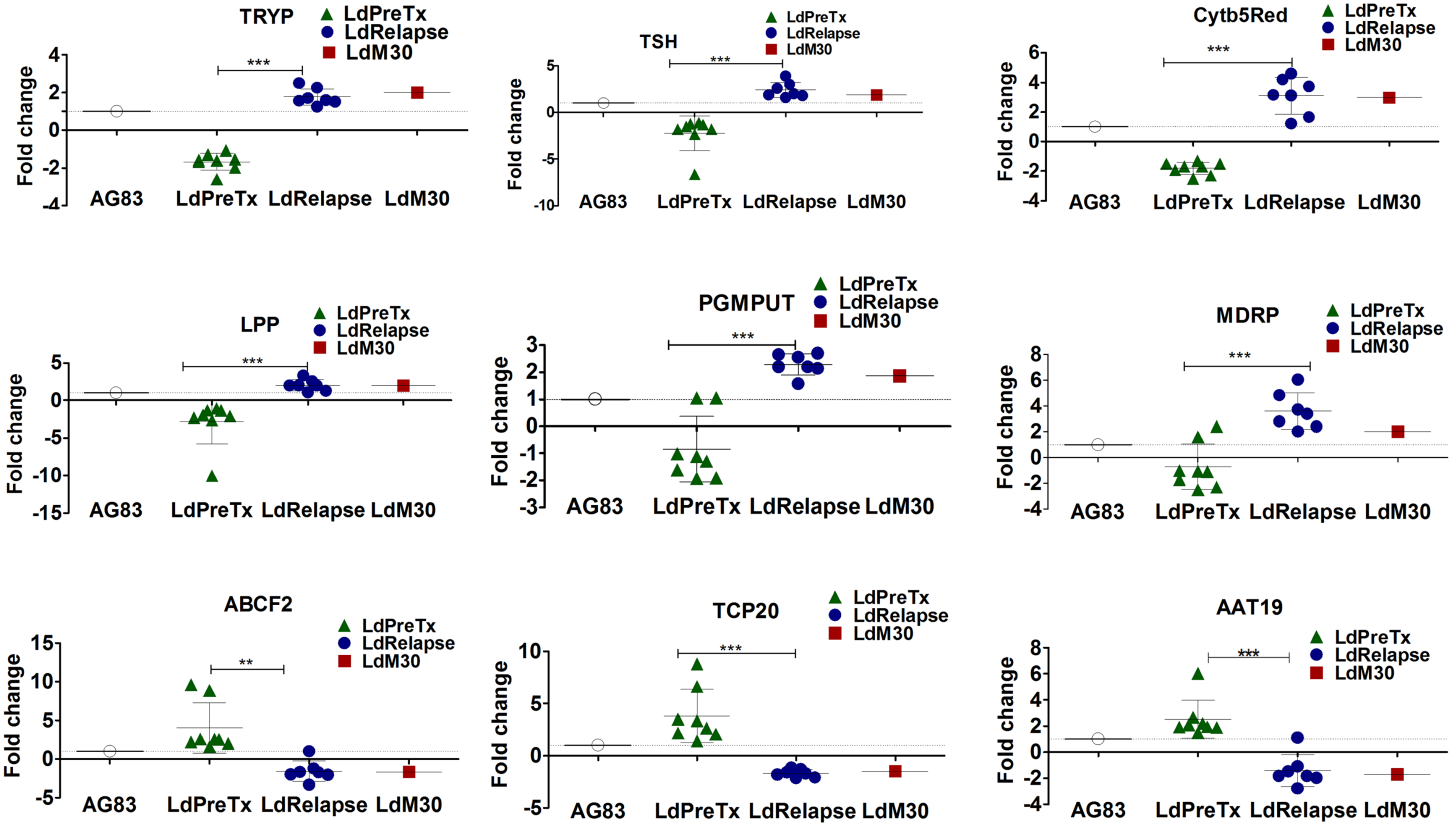
Expression analysis of selected genes by real time PCR in clinical isolates of *L*. *donovani* at pretreatment (LdPreTx), relapse (LdRelapse), LdM30 parasites. Fold change represents expression of target genes normalized to internal control (GAPDH and α-Tubulin) genes and relative to LdAG83. Data represents mean ± SD of two separate assays, each performed in triplicate. Asterisks indicate significance (*P<0.05; **P<0.01; and *** P<0.001).

The genes (n = 9) for expression profiling in clinical isolates were selected based on previous transcriptome studies on MIL sensitive and resistant *L*. *donovani* parasites (Table 1) [12]. The expression levels of selected genes with respect to LdAG83 in clinical isolates (LdPreTx n = 8 and LdRelapse n = 7) and LdM30 (n = 1) are depicted in Fig 3. Based on the fold changes, gene expression levels were designated as increased (N-fold ≥1.5; p<0.05), comparable (N-fold ranging from -1.49 to 1.49) or decreased (N-fold ≤-1.5; p<0.05).

Out of 9 genes evaluated, 6 genes (TRYP, TSH, LPP, Cytb5Red, PGMPUT and MDRP) showed upregulated expression (1.8 to 3.6 fold), while 3 genes (ABCF2, AAT and TCP20) were down regulated (1.6 to 1.9 fold) in LdRelapse group parasites (Fig 3). The expression pattern of the selected genes in LdM30 parasites was consistent with the expression in LdRelapse group parasites.

We observed significant upregulation in the mean expression level ± SD in LdRelapse vs LdPreTx parasite for TRYP (1.78 ± 0.44 vs -1.67 ± 0.46; P<0.01); TSH (2.40 ± 0.76 vs -2.90 ± 2.32; P<0.001); Cytb5Red (3.09 ± 1.16 vs -1.82 ± 0.41; P<0.001); LPP (2.01 ± 0.76 vs -3.20 ± 2.95; P<0.001); PGMPUT (2.30 ± 0.36 vs -1.68 ± 0.39; P<0. 01); MDRP (3.61 ± 1.30 vs -1.58 ± 0.53; P<0.001).

There was significant down regulation in the mean expression level ± SD in LdRelapse vs LdPreTx parasite for ABCF2 (-1.90 ± 0.72 vs 4.22 ± 3.26; P<0.01), AAT (-1.85 ± 0.56 vs 2.70 ± 1.45; P<0.001) and TCP20 (-1.68 ± 0.34 vs 4.70 ± 2.90; P<0.001).

## Discussion

This study revealed modulation in several parasitic factors in LdRelapse isolates in comparison with LdPreTx including (i) increased metacyclogenesis and infectivity (ii) reduced MIL uptake (iii) decreased MIL induced ROS accumulation (iv) increased intracellular thiol content. Analysis of the targeted expression of genes associated with these factors reiterated the above findings in MIL unresponsive clinical isolates of *L*. *donovani*. It is remarkable that the isolates from relapsed cases behaved very similar to the laboratory adapted MIL resistant parasites, however, the magnitude of modulation in gene expression, ROS and thiol levels, drug accumulation was lower in clinical isolates while the infectivity to macrophages was higher. The novelty of the study lies with the investigation of parasitic factors in parasites isolated from PKDL and VL cases at pre treatment and from patients that relapsed after miltefosine treatment. The laboratory generated MIL resistant parasite was used as the reference strain, since well defined miltefosine resistant clinical isolate of *L*. *donovani* are not available.

We observed increased infectivity of LdRelapse parasites to macrophages when compared to LdPreTx group as an important parasitic factor associated with MIL unresponsiveness, unlike the *in vitro* adapted MIL resistant parasite LdM30. High infectivity and metacyclogenesis have been shown to be linked with the high relapse rate in post MIL treated VL cases in Nepal [8].

The reduced uptake of drug, increased efflux and faster metabolism of drug linked with altered plasma membrane permeability have been cited as the most likely mechanisms responsible for development of resistance against miltefosine in *Leishmania* [10–12, 30, 31]. LdRelapse and LdM30 parasites showed significantly lower accumulation of MIL compared to LdPreTx. Further, MIL accumulation in *L*. *donovani* isolates was negatively correlated (r = -0.78) with IC_50_ value. Down regulated expression of phospholipid translocase machinery LdMT and its beta subunit LdRos3 at plasma membrane surface of LdM30 parasites and mutation at these loci result in defective translocation of the drug [12]. Recently, whole genome sequencing approach in clinical isolates of *L*. *infantum*, revealed mutation in MIL transporter genes LiMT and its accessory protein LiRos3 [31]. In our earlier study, we did not observe polymorphism in nucleotide sequence of LdMT-LdRos in LdRelapse, unlike LdM30 which showed two point mutations in LdMT gene sequence [7,12]. This suggests that the reduced uptake of MIL may not be necessarily associated with point mutation in LdMT-LdRos complex in clinical isolates of *L*. *donovani*. The differential accumulation of MIL may be due to low influx or higher efflux as we observed upregulated expression of multidrug resistance like protein (MDRP) and downregulated expression of transporter ABCF2 at mRNA level in miltefosine unresponsive isolates.

ROS mediated apoptosis like cell death in *Leishmania* induced by MIL is widely accepted as one of the mechanisms associated with antileishmanial activity of MIL [15, 32, 33]. To encounter host defence, parasites adopt strategies to overcome the oxidative environment and maintain redox homeostasis. The MIL induced ROS generation pattern was found to be comparable before and after MIL exposure in macrophages infected with LdRelapse and LdM30 parasites. In macrophages infected with LdPreTx parasites the level of ROS was significantly higher (≥2 fold, P<0.05) after MIL exposure. Thus, MIL unresponsive parasites (LdRelapse and LdM30) had survival advantage over LdPreTx.

Thiol metabolism plays an important role in combating drug pressure by suppressing oxidative stress. Although, the individual levels of glutathione and/or trypanothione were not determined, the total intracellular thiol content was found elevated in LdRelapse (1.5 fold) and LdM30 isolates (1.7 fold) compared to LdPreTx.

Trypanothione synthetase (TSH), cytosolic tryparedoxin peroxidase (TRYP) and cytochrome b5 reductase (Cytb5Red) have established role in antioxidant defence and in combating drug induced oxidative stress. We observed increased expression of all the 3 genes in LdRelapse and LdM30 compared to LdPreTx group. Increased intracellular thiol would help parasites to maintain redox homeostasis during MIL pressure.

*L*. *donovani* parasites adopt metabolic reconfiguration by shift in primary carbon metabolism to subvert oxidative stress posed by antileishmanial drug [34]. The key enzymes of glycolytic pathway [12] and glucose uptake remain unaltered during stress in *Leishmania* [34]. We observed increased expression of phosphoglucomutase putative (PGMPUT) in LdRelapse and LdM30 parasites. This increased expression of PGMPUT would increase conversion of glucose-1-phosphate to glucose-6-phosphate, an important metabolic intermediate in glycolysis and pentose phosphate pathway, leading to generation of ATP molecules to meet energy demands during oxidative stress posed by miltefosine exposure.

Lipases play important role in acquisition of host resources for energy metabolism and building blocks for the synthesis of complex parasite lipids important for membrane remodelling [35]. We observed increased expression of gene encoding lipase precursor like protein (LPP) in LdRelapse and LdM30 parasites [12]. This could be another strategy adopted by miltefosine unresponsive *L*. *donovani* parasites to exploit lipid catabolism and use of free fatty acid as an alternate energy source during miltefosine pressure.

Elevated level of amino acids help drug resistant parasite in surviving within parasitophorous vacuoles [36]. A recent study has shown downregulated expression of amino acid permease in MIL resistant *L*. *donovani* parasites [37]. We found downregulated expression of a gene coding for amino acid transporter in MIL unresponsive phenotype (LdRelapse and LdM30) of *L*. *donovani*. A recent study highlighted the downregulated expression of mitochondrial HSP70 in MIL resistant *L*. *donovani* parasites [38]. The chaperones TCP20 responsible for protein folding showed downregulated expression in LdRelapse and in LdM30 parasites as reported earlier [12].

The development of miltefosine unresponsiveness in *L*. *donovani* parasites is a pleotropic phenomenon. The present study revealed that parasites isolated from the cases that relapsed exhibited high infectivity, increased metacyclogenesis, reduced drug accumulation and reconfigured metabolism to overcome the oxidative stress induced during MIL exposure, factors contributing to high relapse rate observed in MIL treated VL and PKDL patients. This study highlighted that the overall changes in parasitic factors investigated here are similar in clinical and laboratory adapted parasites, but possibly, different mechanisms are operative for adaptation in the field.
